# The EU Referendum and Experiences and Fear of Ethnic and Racial Harassment: Variation Across Individuals and Communities in England

**DOI:** 10.3389/fsoc.2021.660286

**Published:** 2021-05-14

**Authors:** Alita Nandi, Renee Reichl Luthra

**Affiliations:** ^1^Institute for Social and Economic Research, University of Essex, Colchester, United Kingdom; ^2^Department of Sociology, University of Essex, Colchester, United Kingdom

**Keywords:** harassment, Brexit, ethnicity, immigrants, discrimination, hate crime, fear, EU referendum

## Abstract

This paper uses nationally representative, longitudinal data to examine experiences and fear of ethnic and racial harassment in public spaces among minorities in the UK, comparing levels of both before and after the 2016 EU Referendum. We do not find an increase in the prevalence of ethnic and racial harassment, but we do find higher levels of fear of ethnic and racial harassment in the period after the Referendum. The increase in fear following the vote was concentrated among more privileged individuals: those with higher levels of education, and those living in less socioeconomically deprived areas with lower levels of previous right-wing party support. We conclude that the Referendum exacerbated already higher levels of perceived discrimination among higher educated minorities while reducing the buffering effect of residence in “safe areas.”

## Introduction

There is emerging evidence that electoral events might influence dominant majority attitudes toward out-group members, including ethnic and racial minorities. There is also substantial evidence that media coverage of politicized events can heighten out-group hostility (Adena et al., 2015), reinforcing existing prejudices and even creating new ones (Gavin, 2018). The heightened salience of outgroup membership and increased hostility toward outgroup members which arises from significant events such as the September 11 terrorist attacks or the Trump campaign has further been shown to increase the prevalence of hate speech (Newman et al., 2020), actual acts of hate crime (Frey, 2020) and discriminatory practices toward ethnic minorities (Rasul and McConnell, 2020).

In this paper, we test whether one such politicized event—the 2016 EU Referendum– increased reports of ethnic and racial harassment (physical or verbal attacks perceived by the victim to be due to their race, ethnicity, nationality, language, accent, dress or appearance) in public places as well as fear of such harassment for both white and non-white ethnic minorities in the UK. We are interested in both the actual experience of harassment as well as fear of such experiences as prior research has shown that both can have detrimental effect on mental health (Nandi et al., 2016, 2020). In addition to improving our understanding of the effect of racially politicized events on outgroup attitudes and discriminatory practices, our aim with this study was to provide an updated account of experiences and fear of ethnic and racial harassment in British society following the EU Referendum vote. The primary debate about the consequences of this event on the British population has been the expected economic costs after leaving the EU, but less attention has been paid to a potential change in the experiences and feeling of safety for ethnic minorities.

An increase in racially motivated hate crimes based on police reports following the EU referendum has been well-documented (Carr et al., 2020; Home Office, 2020). Carr et al. (2020) also show that this increase was unlikely due to increases in reporting alone but rather due to changes in social sanctions against discriminatory attitudes, alongside inflammatory media reporting, that allowed for more actual acts of hate crime. However, as these reports and papers are based on police reports of hate crimes they do not capture milder forms of harassment that would be unlikely to be reported to the police or when reported would be counted as hate incidents and not hate crimes (as these may not constitute a criminal offense). Police reports will also not be able to capture fear of ethnic and racial harassment.

Our first contribution, in this paper, is to provide a more complete picture of potential changes in the environment for England's ethnic minorities following the EU Referendum vote, by examining both harsher and milder forms of ethnic and racial harassment as well as reports of fear of experiencing such harassment. To do this we use data from *Understanding Society:* the UK Household Longitudinal Study (UKHLS), which interviews a nationally representative sample of UK residents every year and asks a sub-sample comprising primarily of ethnic minorities and immigrants a series of questions about various aspects of their experience of harassment including feeling unsafe. As the EU Referendum was on 23rd June 2016, in our analysis we use data collected from January 2015 until early 2019 (fieldwork runs throughout the year).

Our second contribution is providing representative [for England] estimates of these changes from before and after the EU Referendum, by using data from Understanding Society and by using appropriate weights to account for non-response bias. Whereas, estimates based on police reports suffer from reporting bias, as individuals non-randomly select into self-reports of hate crime to the police, our use of a nationally representative sampling frame with appropriate weights results in population level estimates of experiences and fear of ethnic and racial harassment. Third, by considering a longer time-period we argue that this analysis tests for a more general change in tone and incivility following the EU Referendum vote. Finally, as the UKHLS asks a vast array of questions about almost every aspect of a person's life, we are able to measure heterogeneity of this effect, which in turn allows us to test hypothesized mechanisms that could explain the relationship between the EU Referendum and ethnic and racial harassment.

### Background and Hypotheses

There is an established body of work that documents a relationship between electoral events such as the EU Referendum or Brexit vote and changes in attitudes and behaviors (Leduc, 2002). One such behavior that has recently received substantial research attention is racially motivated hate crime and hate incidents. A hate crime is defined by British law as “Any criminal offense which is perceived by the victim or any other person, to be motivated by hostility or prejudice based on a person's race or perceived race; religion or perceived religion; sexual orientation or perceived sexual orientation; disability or perceived disability and any crime motivated by hostility or prejudice against a person who is transgender or perceived to be transgender” (Metropolitan Police, 2020). A hate incident is defined as any incident that is perceived as motivated by one of these characteristics, and while these may not be criminal in character, can still be reported and documented by the police. Hate incidents are not included in the statistics on hate crimes published by the Home Office (e.g., see Home Office, 2020). In this paper, we examine the pre- and post-Referendum prevalence of a related concept, ethnic and racial harassment, through the perspective of the victim; that is through self-reports of experiences of verbal or physical attacks in public places that the victim attributes to their ethnicity, religion, nationality, language, accent, dress or appearance. Our measure of these incidents does not include any reference to a crime and so need not be severe enough to constitute a criminal offense and hence a hate crime. In taking the victim's perspective, we are also interested in how Brexit may have influenced feelings of vulnerability in public spaces by white and non-white ethnic minorities, which we measure as reports of feeling unsafe or avoiding public places because of these same characteristics.

Theories of social or group-based identities, developed by social psychologists, have long established the existence of favorable attitudes and behavior toward in-group members and discriminatory practices toward out-group members (Tajfel, 1981). Sociologists (Bobo, 1999) and economists (Akerlof and Kranton, 2000) discuss how these individual preferences, produced and strengthened by group-based identities, operate in the aggregate to shape ethnic inequality in life chances, as well as perpetration of and exposure to discriminatory acts. In times of increased competition for scarce resources, as well as opportunities and events that heighten the salience of social identities such as the EU Referendum, these prejudicial attitudes may strengthen, leading to increases in hate crimes and incidents [for a review see (Luthra and Nandi, 2020)].

Populist electoral events and parties increase the salience of in-group identities by framing an “in-group” of the “people” against a notion of a corrupt “elite,” an in-group that can be based on cultural, political or class basis (Kriesi, 2014). The EU Referendum has been widely defined as a populist electoral event, as the Leave campaign portrayed leaving the EU as the preservation of British sovereignty against Brussels elites and built on a nativist turn away from multiculturalism and toward “British values” (Cutts et al., 2011). The campaign was highly polarizing and increased the saliency of British national identities as well as the salience of immigrants as out-group members in the UK (Henderson et al., 2017). The campaign squarely placed EU membership as a cause of both cultural and economic decline in Britain (Davis et al., 2019), fomenting popular perceptions of competition for scarce resources with “uncontrolled” migration from the EU serving as the primary mediator of this threat.

Whether this increase in saliency directly led to increases in ethnic and racial harassment in the UK remains unclear. On one hand, official hate crime data from the UK Home Office shows a sharp increase in reports of ethnically motivated hate crime in the lead up to and directly following the Referendum, and this increase does not appear to be explained by changes to reporting alone. Using hate crime data aggregated at local levels, researchers find that the post-Referendum increase is higher than would be expected based on general demographic and seasonal reporting models, and that it is higher in areas that voted more strongly in favor of leaving the EU. The authors of these studies argue that the increase can be linked to a general shift in attitudes toward out-group members and that Brexit support signaled tolerance for racially motivated abusive behavior (Albornoz et al., 2020); they also find that media coverage of both hate crime and the Referendum itself are associated with small spikes in hate crime reporting (Carr et al., 2020). These studies, on the other hand, also find that the spike in hate crime reporting is generally short lived—concentrated in the quarter following the Referendum. This fits previous research which finds that hate crimes are often linked to an antecedent event, conceptualized as a *reaction* when a highly publicized event stokes anti-outgroup sentiment, and are often short in duration and strongest when a specific group is highlighted (King and Sutton, 2013).

While police reports of hate crime cover the more egregious acts of hostility against minorities, research which relies on this data alone naturally misses more minor forms of harassment and it is possible that increases in hate crime are short-lived and do not reflect more general change in majority attitudes and behaviors toward ethnic minorities. For instance, research which attempts to address whether the spike in police reported hate crimes is also mirrored in more population level self-reported attitudes is mixed. Schwartz et al. (2020) demonstrate that among those who voted Leave, anti-immigrant sentiment actually softened following the EU Referendum due to a greater sense of control, and that fears of appearing xenophobic or racist softened anti-immigrant sentiment among both Leavers and Remainers. Using experimental evidence designed to uncover hidden attitudes due to social desirability bias, other research has shown that while overt expressions of prejudice against for instance Muslim immigrants had declined following the EU Referendum vote, more covert measures of discrimination remained (Creighton and Jamal, 2020).

Thus, the evidence on whether this campaign actually *caused* an increase in xenophobic and racist sentiment is equivocal, and current research linking the EU Referendum vote to hate crime specifically thus far focuses on police reports, which include only those forms of ethnic and racial harassment which constitute a criminal offense and which undercount the actual incidence of hate crime and hate incidents. For instance, the number of racially motivated hate crimes reported to the police increased from around 49,000 in 2015/16 to around 71,000 in 2017/18 while the estimated number of racially motivated incidents and hate crimes during this period based on the Crime Survey of England and Wales (CSEW), a nationally representative survey of victimization, was around 100,000 per year (Home Office, 2018). Even the CSEW uses at best a restricted definition of ethnic and racial harassment, as respondents are first asked whether they have been a victim of a *crime* and are only then followed up with a question on whether this crime was racially motivated. They will thus not report milder forms of harassment such as being insulted. To illustrate this point, earlier comparisons of CSEW estimates of hate crime with the more expanded definition of ethnic and racial harassment that we use here show that the (weighted) estimate of the proportion of ethnic minorities who report being the victim of at least one racially motivated crime in the past year during 2008–2016 based on CSEW is 2.3%, while the (weighted) estimate of the proportion of ethnic minorities who reported being physically or verbally attacked because of their ethnicity, religion or nationality in a public place in the past year during 2009–2015 is 10–20% for men and women of different ethnic groups (Nandi and Luthra, 2016).

In terms of the lived experiences of immigrants and ethnic minorities in the UK, it is also important to consider how the EU Referendum might have increased both harsher and milder forms of ethnic and racial harassment as well as their perceptions of hostility of the majority group toward them and consequently their own feelings of vulnerability. For this we need a victim-centered approach, examining not only hate crime as reported to the police, but also general reports of hostility. Most of this work has taken the form of qualitative interviews, which demonstrates increased feelings of unwelcome and vulnerability in the run-up to and following the EU Referendum. The vote made visible existing economic and social vulnerabilities among EU migrants as well as created new ones (Burrell and Schweyher, 2019), and qualitative accounts document how the EU Referendum exposed EU migrants to common experiences for third-country migrants which may have been invisible to them before—for instance the experience of monitoring from their employers (Manthorpe et al., 2018; Luthra, 2021), navigating bureaucratic requirements to secure legal permanent residency or citizenship (McGhee et al., 2017), and fraught interactions with the Home Office (Yuval-Davis et al., 2017). The vote also created, or heightened, feelings of hostility and unwelcome, with respondents reporting an increase in awareness of anti-immigrant hate crimes (Rzepnikowska, 2019) as well as a disruption of feelings of belonging and sense of personhood among long-standing EU residents in the UK (Guma and Dafydd Jones, 2019).

Moreover, there is some evidence that negative press coverage of some out-groups, in this case EU immigrants, can also “spillover” into greater hostility toward other minorities. For example, US researchers have demonstrated that negative media portrayals linking Islamophobia and immigration following the 9/11 attacks (Romero and Zarrugh, 2018) resulted in longer prison sentences for minority group members perceived as immigrants (Rasul and McConnell, 2020). Analysis of a recent survey of mental health among migrants of all origins in the UK demonstrate how an increase in minority stress and social stigma, mediated via increased experiences of discrimination following the Referendum, resulted in worse mental health for both EU and non-EU migrants (Frost, 2020). Similar to work with police data, the reported increases in discrimination and mental ill health were higher in areas with more Leave voters. Given existing evidence that suggests a positive relationship between events which increase the saliency of in-group identities and heighten feelings of animosity toward and competition with out-group members, we further expect that the EU Referendum may increase feelings of unwelcome and exposure to ethnic and racial harassment among other out-groups in the UK, namely ethnic and racial minority immigrants and their descendants.

*H1: The EU Referendum will be associated with an increase in reports of experience and fear of ethnic and racial harassment for EU immigrants as well as non-white immigrants and their descendants*.

Beyond a general increase in ethnic and racial harassment and feeling unsafe following the EU Referendum among minorities in the UK, we might also expect both individual and community level variation in this association. Several scholars have noted how “diverse histories and geographies of marginality and privilege shape responses to and impacts of Brexit” (Botterill et al., 2019, p. 2). Many of these scholars have pointed to individual level characteristics, such as labor market position, social class (Lulle et al., 2019) and race (Benson and Lewis, 2019), to argue that vulnerabilities to the EU Referendum can align with existing stratification.

Previous research on the fear of victimization and actual victimization in the general population uncovers a paradox in terms of age and gender: although older individuals and women generally report lower levels of victimization then men and younger individuals, women and older individuals still report higher levels of *fear* of crime (Stafford and Galle, 1984; Smith and Tortensson, 1997). These scholars point toward the greater physical vulnerability of women (Smith and Tortensson, 1997) and older individuals (De Donder et al., 2005), as well as underreporting and discounting of fear by men (Sutton and Farrall, 2005), to understand this paradox. Specifically in terms of discrimination and racially motivated crime, a body of work also demonstrates a further paradox in that minorities of higher socio-economic standing also report higher rates of discrimination (de Vroome et al., 2014; Steinmann, 2019), likely for the reason that they have higher expectations of fair treatment and their higher socioeconomic position puts them in greater contact with the majority group. Evidence of this “integration paradox” has similarly been demonstrated in the UK in work that finds higher reports of ethnic and racial harassment among ethnic minority men, those who are younger, and are more highly educated (Nandi and Luthra, 2016), but that feelings of unsafety are primarily stratified by sex, with women reporting higher levels of fear of ethnic and racial harassment. Drawing on this previous research, we might expect that the EU Referendum will *exacerbate* existing inequalities in exposure to ethnic and racial harassment and feelings of unsafety:

*H2: The EU Referendum will be associated with an increase in reports of ethnic and racial harassment among younger and male ethnic minorities, but an increase in reports of fear of ethnic and racial harassment among older and women ethnic minorities*.

*H3: The EU Referendum will be associated with an increase in reports of experiences and fear of ethnic and racial harassment among ethnic minorities of higher socio-economic status*.

Finally, although spillover effects are noted in the literature and media coverage of the EU Referendum often conflated the Referendum with immigration policy in general (Walter, 2019), we do know that free movement and Euroscepticism were particularly targeted in the Leave campaign immediately preceding the vote (Morrison, 2019). Thus, any post-Referendum increase in experiences or fear of ethnic and racial harassment may be stronger for those of European origins:

*H4: The EU Referendum will be associated with a higher increase in experiences and fear of ethnic and racial harassment for European immigrants than for other minorities*.

Hypotheses 2–4 anticipate individual level variation in the association between the EU Referendum, ethnic and racial harassment, and fear of ethnic and racial harassment. However, there may be variation in these associations across different communities as well. First, criminological research on both hate crime and fear suggest that they should be higher in areas that are more economically deprived. In terms of neighborhood deprivation and fear of crime, a well-established perspective on the meso- or ecological determinants of fear of crime concerns the role of “social disorganization”—including exposure to incivilities, physical infrastructure, visible signs of disorder, as well as actual local victimization rates—at the local level as being a major determinant of fear of crime. These factors are often proxied by the index of multiple deprivation, which is an aggregated measure of many forms of socioeconomic vulnerability that are correlated with the physical environment and exposure to disorder and incivilities at the neighborhood level. Neighborhood level characteristics often moderate relationships with fear of crime (Brunton-Smith and Sturgis, 2011) and thus it follows that they may moderate fear response to the Referendum vote as well.

Beyond perceptions of crime, criminological research also documents a clear association between area deprivation and crime (Peterson et al., 2000). Drawing on General Strain Theory (Agnew, 2014), crime is expected to be higher in areas where there is a concentration of individuals experiencing multiple barriers to achieving desired social goals such as financial security or respect, where social control is low, and where there is greater opportunity for the social learning of crime. Related sociological and economic theories of realistic conflict similarly suggest that living in deprived areas gives rise to a greater sense of inter-group conflict over scarce resources (Sniderman et al., 2004; Zárate et al., 2004), which also should lead to higher levels of hate crime and discrimination. Current evidence using police reports of hate crime (Home Office, 2020) as well as ethnic and racial harassment as we define it here (Nandi et al., 2016) corresponds to these theoretical expectations, consistently finding higher rates of victimization in more deprived areas.

Second, in addition to general indicators of deprivation, we might also expect that the ethnic composition of the local area will influence exposure to harassment as well as fear. Previous research has established that the risk of ethnic and racial harassment is lower in areas with higher proportions of ethnic minorities (Dustmann et al., 2011). Whether general deprivation and co-ethnic composition also means an increased negative impact of the EU Referendum is however less clear. Research on the effect of perceived threat heightening events such as the EU Referendum on hate crime turn instead to more proximate measures of out-group hostility as potential community level moderators, which can be positively correlated with deprivation but are conceptually distinct. For instance, emerging research about the effect of the EU Referendum on hate crimes using police reports show higher increases in hate crime in areas that had a higher proportion of Leave voters (Carr et al., 2020). These authors suggest that the EU Referendum made what was likely somewhat hidden (due to social norms) anti-immigrant bias public, changing the social norms around expression of anti-immigrant sentiment and thus lowering the informal costs of committing hate crime. Qualitative research on changes in perceptions of hostility following the EU Referendum also find community level variation in its effects, for instance experiences of increased vulnerability in neighborhoods perceived as dominated by white working class “others” (Rzepnikowska, 2019) or in devolved regions such as Wales (Guma and Dafydd Jones, 2019) as contrasted to the more diverse and accepting “Eurocity” of London (Favell, 2011; Lulle et al., 2019). Existing research thus leads us to expect a more pronounced EU Referendum effect in areas which are more deprived, have a lower proportion of co-ethnic residents, and have stronger support for anti-immigrant sentiment.

*H5: The EU Referendum will be associated with a stronger increase in experiences and fear of ethnic and racial harassment in deprived areas, in areas with a lower proportion of co-ethnic residents, and in areas with previous right-wing electoral support*.

## Data and Methods

### Methods

We estimate models of the likelihood of reporting experiences and fear of ethnic and racial harassment using logistic regression. We use longitudinal weights to account for differential selection probability and non-random attrition and estimate standard errors after taking into account the complex survey design which is clustered and stratified. As the models include the general health question which was asked in a self-completion questionnaire we use self-completion longitudinal weights that account for non-random response to this part of the survey. The statistical software package we used was STATA version 16. We estimated the models using the *logit* command with the svy prefix suite of commands that allow accounting for weights and sample design. The average marginal effects (AMEs) reported in the tables are produced using the post-estimation command *margin*, and in models where a variable was interacted with the EU Referendum dummy, *contrast* was used to test the difference in AME for different categories of the variable with the AME of the reference category reported as a reference point. For the categorical variables we use here, AME are the average (across the sample) increase or decrease in an estimated probability of the event for different categories of the variable as compared to a reference category. In non-linear models like logit, this is a better way to show the effect of a variable on the outcome particularly when including interactions (Norton and Ai, 2003).

First, we estimate these models only for the pre-Referendum period to identify how experiences of ethnic and racial harassment and fear of ethnic and racial harassment was distributed across the population. Second, we estimate these models for the pre and post EU Referendum period including a post-Referendum indicator (without any controls) to estimate change from the pre- to post-Referendum period. Third, we re-estimate these models with the variables included in the pre-Referendum models interacting with each variable separately to identify heterogeneity in changes in experiences and fear of ethnic and racial harassment. As the EU Referendum was a random event unrelated to any individual or subnational characteristics, we did not need to include any controls in our models to examine the difference in experiences and fear of ethnic and racial harassment post-Referendum, our first hypothesis. However, to assess hypotheses H2-H5, which posit heterogeneity in changes in experiences and fear of ethnic and racial harassment post-Referendum, we interacted the EU Referendum indicator with several individual level and community level characteristics which were all included as controls to be able to estimate the net effects.

Fourth, to test for the robustness of our findings, we also allowed for temporal variation in post-Brexit changes to experiences or fear of ethnic and racial harassment. First, as previous research on changes in attitudes or discriminatory acts following electoral events generally finds that such changes are short-lived (Cappiali et al., 2018; Schilter, 2020), we tested for changes in experiences of and fear of ethnic and racial harassment across different periods following the EU Referendum. We partitioned the post referendum observation window into four 6 months periods (smaller time periods would have resulted in very small sample sizes) and assessed for variation in post-Brexit changes across time. The four post referendum periods are July-December 2016, January-June 2017, July-December 2017, January-June 2018, July-December 2018 (this includes a few interviews in 2019). Finally, to allow for the fact that our measures of experience and fear of ethnic and racial harassment refer to experiences in the previous 12 months, as further sensitivity test, we also excluded survey respondents who replied to the question within 12 months of the Referendum vote (June 2016), so that the reports of experiences and fear of harassment included in the analysis *only* refer to the post-Brexit period.

### Data

We use data from *Understanding Society* (University of Essex, 2020)^1^, a longitudinal household survey that began in 2009, primarily drawing on waves nine and nine which bracket the 2016 EU Referendum over the period 2015–2018^2^. The sample is comprised of a nationally representative sample of around 26,000 households (General Population Sample, GPS) and an ethnic minority boost sample (EMBS) of 4,000 households where at least one person was from an ethnic minority background. In 2015, an Immigrant and Ethnic Minority boost sample (IEMBS) of around 2,500 households was added. A household qualified for inclusion if at least one household member was born outside the UK or was from an ethnic minority background (same criterion as for the EMBS). Households selected into these samples are randomly assigned to monthly or quarterly samples which determines their interview and fieldwork period and every year they are interviewed in that specific month or quarter (Lynn, 2009). While sample members can choose when they would like to be interviewed, the dates are constrained within their randomly assigned months or quarters.

Every year respondents in the sampled households who are 16 or above are asked questions about different aspects of their lives including parental and family background, socio-demographic characteristics, education, labor market experience, income and wealth, health and wellbeing, attitudes, values and beliefs. A sub-sample, known as the “extra 5 min sample” comprising primarily ethnic minorities and immigrants, are asked 5 min worth of additional questions. In Waves seven and nine, covering the interview period 2015–2018, this includes questions about experiences of harassment. See (McFall et al., 2020) for further details about the screening questions, the design for the boost samples and the composition of the extra 5 min sample.

As the goal of this paper is to examine changes in ethnic and racial harassment and fear among ethnic minorities, we exclude all respondents who identify as White British from our sample.

Using the residential location of the sample members, we match the survey data with Census 2011 data on number of adults from different ethnic groups at the Lower Super Output Area (LSOA). LSOAs are areas with around 650 households and 1,500 individuals and we consider these areas to be proxies for neighborhoods of respondents in the study. We also match the survey data with the 2010 Index of Multiple Deprivation (IMD) at the LSOA level. The English IMD 2010 is computed at the LSOA level by combining levels of seven domains of deprivation (income, employment, health and disability, education skills and training, barriers to housing and other services, crime and living environment) using 38 individual measures with each domain weighted appropriately. As this measure is not compatible across countries of the UK, we restricted the analysis to England where 94% of UK's ethnic minorities live^3^. Finally, using data from the 2015 General Election data at the parliamentary constituency level available from the Electoral Commission^4^, we match the proportion of the electorate who voted for populist right wing parties—UK Independence Party or the British National Party (BNP) (Cutts et al., 2011; Goodwin, 2011)—in the 2015 general election as a proxy for existing hostility from a period prior to the Referendum campaign.

### Variable Descriptions

#### Ethnic and Racial Harassment and Feeling Unsafe

The harassment module includes four sets of questions which follow the same pattern. Each set starts with one of these questions:

- “In the last 12 months, have you felt unsafe in any of the places listed on this card? If so, which ones?”
- “In the last 12 months, have you avoided going to or being in any of the places listed on the card? If so, which ones?”
- “In the last 12 months, have you been insulted, called names, threatened or shouted at, in any of the places listed on this card? If so, which ones?”
- “In the last 12 months, have you been *physically attacked* in any of the places listed on this card? If so, which ones?”

If the respondent then chose one of the places listed on the card (Home, School, Colleges or Universities, Work, Streets, Shops, Public transport, Stations, Taxis, other places) they are asked a follow up question about the reasons they think this happened. The possible reasons they could choose from were (they could choose more than one reason):

*Your* sex, age, ethnicity, sexual orientation, health or disability, nationality, religion, language or accent, dress, or appearance, none of the above (spontaneous), other reason.

We defined a person to have experienced ethnic and racial harassment if they reported being physically attacked or insulted in at least one place other than their home, school, university or place of work and if they chose ethnicity, nationality, religion, language or accent, or dress or appearance as one of the reasons. It is clear from the question text that our definition of ethnic and racial harassment does not require the incident to be identified as a criminal offense.

We defined a person to feel unsafe for fear of ethnic or racial harassment if they reported feeling unsafe in at least one (public) place other than their home, school, university or place of work and if they chose ethnicity, nationality, religion, language or accent, or dress or appearance, as one of the reasons. Although “dress or appearance” and “Language or accent” are less explicitly tied to race and ethnicity, we included these characteristics as they are more salient for European origin respondents.

#### The EU Referendum

We compare experiences and fear of ethnic and racial harassment before and after the EU Referendum by including an indicator variable which takes on the value 1 if the respondent was interviewed after 23 June 2016 and 0 otherwise.

#### Individual and Community Level Moderators

##### Age and Sex

Age is measured in years as of the date of the interview. Dates of birth and sex are as reported by the respondent or their household members if not self-reported. To test H2, these two variables are interacted with the Referendum indicator.

##### Socioeconomic Status

We include two measures of socioeconomic status. The first is educational attainment, dichotomized as those with and without a university level degree or higher. We also included a category for those with missing education information. In the wave that the IEMB sample was added (Wave 6, 2015), adult respondents who had received their qualification outside the UK were asked an educational qualification question with a different set of response categories. As a result their educational qualification variable was missing unless they acquired a qualification in the UK since then. In an alternate version we coded those who had chosen categories closest to a “first degree or higher” as having received a degree. In this harmonised version of the degree variable there were only around 40 cases with missing value for the degree variable. The second is equivalized gross household income, the gross monthly income reported at the household level, equalities for household size and composition with the modified OECD equivalence scale^5^ and entered into the model in quintiles. These two interactions are used to test H3.

##### Ethnic and Immigration Background

We exclude those who identify as White—British, English, Scottish, Welsh or Northern Irish. To address potential heterogeneity in the EU Referendum effect for those most targeted in xenophobic rhetoric surrounding the Leave campaign (H4), we identify those who were born in the original 15 EU member states and those born in the more recent accession countries (Czech Republic, Estonia, Hungary, Latvia, Lithuania, Poland, Slovakia and Slovenia, who joined in 2004, and Bulgaria and Romania, who joined the EU in 2007). We also separate all non-white ethnic minority individuals into those who were born in the UK and those born outside as the second generation may have different experiences and perceptions of harassment and fear. The remaining sample are included into a residual “other category.” This group is comprised mostly of “White: Other” respondents born in non-EU countries (~65%), and “White: Other” respondents born in UK (~20%).

##### Local Area Characteristics

Local area deprivation is measured using the Index of Multiple Deprivation at the LSOA level as described in the data section above. The continuous measure is categorized into quartiles. Previous right-wing electoral support is measured as the voter share at LSOA level for the BNP and UKIP parties in the 2015 general election, as described above. We categorized this continuous measure into quartiles. Finally, co-ethnic composition is measured as the proportion of residents reporting the same ethnic group (measured by the 2011 Census ethnic group question which was asked of Understanding Society respondents as well) as the respondent at the LSOA level using data from the 2011 Census. As White British residents make up to 80% of England's population, they mostly live in areas with other White British residents. So, categorizing this measure for all respondents would have resulted in ethnic minorities living mostly in the lowest quartiles. Thus, to be able measure variation across areas in our sample of ethnic minorities we categorized this continuous measure into quartiles by only considering the areas with ethnic minority respondents (unlike for the local area deprivation and right wing support measures).

##### Controls

We also include controls for general health, full-time student status and partnership status in these models. General health is measured via self-reported health which asks respondents “In general, would you say your health is…” and we dichotomize the results, with those reporting excellent, very good or good health scoring one and those with fair or poor health scoring zero. The partnership status variable is a 0–1 indicator for whether the respondent is married or cohabiting vs. those who do not live with a partner. Finally, we include a 0–1 indicator for respondents who are full-time students.

## Results

### Descriptive Statistics

The sample comprises of ethnic minorities living in England who were interviewed between 2015 and 19. In Table 1 we show the descriptive statistics for our sample, before and after the EU Referendum. The first two columns include unweighted proportions and thus describe the sample. The third and fourth columns show weighted proportions and thus provide population estimates before and after the EU Referendum. Weighted estimates show a higher proportion of ethnic minorities reporting fear of ethnic and racial harassment after EU Referendum (7 vs. 9%, and this difference was statistically significant) but no difference in report of experiences of ethnic and racial harassment (5%). We find one or two percentage point differences in pre and post Brexit compositions for most individual and area level characteristics, but none of these differences are statistically significant except for the one percentage point decrease in the proportion of “other” immigrants.

**Table 1:** Descriptive statistics.

	**Unweighted**		**Weighted**		
	**Before**	**After**	**Before**	**After**	***p*-value of test of difference**
	**The EU Referendum**				
Physically or verbally attacked in public places in the past 12 months due to one's ethnicity, religion, nationality, language, accent, dress or appearance					
No	95%	94%	95%	95%	0.714
Yes	5%	6%	5%	5%	
Felt unsafe in public places the past 12 months due to one's ethnicity, religion, nationality, language, accent, dress or appearance					
No	92%	88%	93%	91%	0.002
Yes	8%	12%	7%	09%	
Gender					
Men	41%	43%	44%	46%	0.158
Women	59%	57%	56%	54%	
Age group					
16–19 years	8%	8%	10%	9%	0.457
20–29 years	14%	16%	16%	17%	0.507
30–39 years	21%	21%	23%	23%	0.988
40–49 years	23%	22%	23%	22%	0.559
50–59 years	17%	16%	15%	14%	0.903
60+ years	17%	16%	14%	15%	0.258
Highest educational qualifications					
No college degree	62%	60%	61%	58%	0.056
College degree or higher	32%	32%	35%	37%	0.200
Information missing	6%	8%	4%	5%	0.337
Equivalised gross household income					
Lowest quintile	21%	20%	18%	15%	0.344
2nd quintile	20%	20%	20%	18%	0.414
3rd quintile	20%	20%	19%	19%	0.829
4th quintile	17%	19%	17%	20%	0.125
Highest quintile	22%	22%	26%	27%	0.474
Ethnic and immigration background					
Born in EU15	8%	6%	13%	11%	0.173
Born in A2A8	5%	6%	8%	10%	0.143
Non-white ethnic minorities, born in UK	29%	31%	23%	24%	0.430
Non-white ethnic minorities, born outside UK	47%	47%	38%	38%	0.959
Other	11%	9%	18%	16%	0.018
Deprivation level in the neighborhood or LSOA					
Lowest quartile	15%	14%	24%	23%	0.485
2nd quartile	19%	18%	26%	24%	0.112
3rd quartile	27%	28%	22%	24%	0.347
Highest quartile	39%	41%	27%	30%	0.174
Proportion of co-ethnic residents in the neighborhood or LSOA					
Lowest quartile	22%	23%	30%	32%	0.490
2nd quartile	26%	22%	27%	25%	0.401
3rd quartile	21%	24%	17%	18%	0.465
Highest quartile	31%	30%	26%	25%	0.568
Proportion of voters in the parliamentary constituency who voted for UKIP or BNP in the 2015 General Elections					
Lowest quartile	34%	34%	29%	30%	0.724
2nd quartile	30%	28%	25%	25%	0.926
3rd quartile	20%	18%	26%	25%	0.391
Highest quartile	16%	20%	19%	20%	0.762
Married or cohabiting?					
No	40%	40%	40%	41%	0.658
Yes	60%	60%	60%	59%	
General health					
Fair or poor	19%	19%	16%	16%	0.884
Food, very good or excellent	81%	81%	84%	84%	
Full-time student?					
No	90%	89%	88%	88%	0.719
Yes	10%	11%	12%	12%	
**Number of observations**	3,004	7,513	3,004	7,513	

*Data: Understanding Society (Waves 7 and 9, 2015–2019)*.

### Prevalence of Experiences and Fear of Ethnic and Racial Harassment Before the EU Referendum

In Table 2, we present results from estimation of models of ethnic and racial harassment (respondent reported being physically or verbally attacked in a public place in the last 12 months and attributed this to their ethnicity, religion, nationality, language or accent, dress or appearance) among England's ethnic minorities in the pre- EU Referendum period (2015-−23rd June 2016). We find that in this period, non-white ethnic minorities were more likely to report experiencing ethnic and racial harassment: UK born non-white minorities were 5% points more likely, and foreign born non-white minorities 3% points more likely, to report ethnic and racial harassment than EU15 minorities in the period prior to the EU referendum vote (although the difference for UK born minorities is not statistically significant). In line with previous research based on data from the late 1990s and 2009–2015 (Dustmann et al., 2011; Nandi et al., 2016), we find that ethnic minorities living in areas with a higher proportion of co-ethnic residents report lower levels of ethnic and racial harassment. As expected, we find that ethnic minorities living in areas with a higher proportion of right wing voters, specifically the highest quartile, are more likely to experience ethnic and racial harassment (although this difference is not statistically significant). This is also consistent with findings by Nandi and Luthra (2016) for the period 2009–2015.

**Table 2:** Average marginal effect (AME) of ethnic and racial harassment and fear of such experiences *before* the EU Referendum.

	**Ethnic and racial harassment^a^**		**Fear of ethnic and racial harassment^b^**	
	**AME**	***p*-values**	**AME**	***p*-values**
Gender (Ref: Men)				
Women	−0.01	0.451	0.02^*^	0.020
Age group (Ref: 16–19 years)				
20–29 years	0.02	0.120	−0.01	0.750
30–39 years	0.03	0.188	0.06^*^	0.034
40–49 years	0.03	0.236	0.05	0.088
50–59 years	0.03	0.503	0.03	0.238
60+ years	−0.01	0.616	−0.01	0.837
Highest educational qualifications (No college degree)				
Received college degree or higher	−0.00	0.982	−0.00	0.794
Information missing	−0.02	0.469	−0.05	0.063
Equivalised gross household income quintiles (Ref: Lowest)				
2nd quintile	−0.01	0.833	0.01	0.739
3rd quintile	0.01	0.733	0.05^***^	0.003
4th quintile	−0.05^*^	0.015	0.01	0.543
Highest quintile	−0.04	0.081	0.01	0.431
Ethnic group (Ref: Born in EU15 countries)				
Born in A2A8	0.01	0.638	0.08^***^	0.001
Non–white ethnic minorities, born in UK	0.05	0.096	0.10^***^	0.000
Non–white ethnic minorities, born outside UK	0.03^*^	0.033	0.07^***^	0.000
Other	0.00	0.821	0.02	0.190
Deprivation level in neighborhood or LSOA (Ref: Lowest)				
2nd quartile	−0.00	0.890	0.01	0.382
3rd quartile	0.01	0.697	0.05^***^	0.000
Highest quartile	0.01	0.644	0.08^***^	0.000
Proportion of co–ethnic residents in neighborhood or LSOA (Ref: Lowest)				
2nd quartile	−0.03	0.294	−0.01	0.542
3rd quartile	−0.04^*^	0.015	−0.04^*^	0.023
Highest quartile	−0.05^***^	0.005	0.02	0.517
Proportion of voters in the parliamentary constituency who voted for UKIP or BNP in the 2015 General Elections (Ref: Lowest quartile)				
2nd quartile	−0.01	0.781	0.05^***^	0.006
3rd quartile	−0.01	0.767	0.07^***^	0.000
Highest quartile	0.04	0.120	0.04^*^	0.029
**Number of observations**	3,004		3,004	

a *Physically or verbally attacked in public places in the past 12 months due to one's ethnicity, religion, nationality, language, accent, dress or appearance;*

b *Felt unsafe in public places the past 12 months due to one's ethnicity, religion, nationality, language, accent, dress or appearance*.

*Models estimated using data from Understanding Society Wave 7 (2015–2016) using logit with longitudinal (self-completion) weights and standard errors estimated after accounting for complex survey design; controls included are general health, FT student and partnership status; + p < 0.10*,

* *p < 0.05*,

** *p < 0.01*,

*** *p < 0.001*.

We next estimated models of feeling unsafe in public places attributed to one's ethnicity, religion, nationality, language or accent, dress or appearance during the pre- EU Referendum period (See the right-hand columns of Table 2). We find that ethnic minority women and those living in more deprived areas are more likely to report fear of ethnic and racial harassment. We also find that compared to migrants from EU-15 countries, migrants from A2 and A8 countries are more likely to report feeling unsafe although we did not find a statistically significant difference in their reporting of ethnic and racial harassment. Non-white ethnic minorities are also more likely to report feeling unsafe than EU15 migrants, as we find to be the case for reporting of ethnic and racial harassment. As with actual experiences, we find that ethnic minorities living in areas with lower proportion of co-ethnic residents and higher proportion of right-wing voters are more likely to feel unsafe.

### Changes in Experiences and Fear of Ethnic and Racial Harassment After the EU Referendum

In Table 3, we report the estimated difference in experiences and fear of ethnic and racial harassment after the Referendum for all ethnic minorities and by different sub-groups. To show the difference in the effect of the Referendum for different sub-groups, we include interactions of the Referendum indicator with each of the variables identifying different subgroups (one at a time) and the AMEs in this table are the AME of Brexit for each category of the variable being interacted. We test whether any change in experiences or fear of ethnic and racial harassment after the EU referendum varied across individuals of different characteristics or for those residing in different types of localities. We report the degree of statistical significance of the test of these differences by sub-groups vis-à-vis the reference category.

**Table 3:** Average marginal effect (AME) of the EU Referendum on ethnic and racial harassment and fear of such experiences (for the whole sample, and separately by sub-groups).

	**Ethnic and racial harassment^a^**			**Fear of ethnic and racial harassment^b^**		
	**AME**	***p*-value**	***p*-value of difference with the reference category**	**AME**	***p*-value**	***p*-value of difference with the reference category**
All	<0.001	0.970		0.03^***^	0.005	
**for different sub-groups**						
Gender						
Men (reference category)	−0.01	0.524		0.03^*^	0.014	
Women	0.01	0.657	0.442	0.02	0.073	0.462
Age group						
16–19 years (reference category)	0.01	0.535		0.04	0.316	
20–29 years	0.01	0.706	0.809	0.06^***^	0.005	0.403
30–39 years	<0.01	0.982	0.643	<0.01	0.962	0.429
40–49 years	−0.01	0.318	0.285	0.02	0.316	0.680
50–59 years	−0.01	0.833	0.596	0.03	0.208	0.981
60+ years	0.01	0.416	0.739	0.03	0.066	0.499
Highest educational qualifications						
No college degree (reference category)	−0.02	0.113		0.01	0.508	
Received college degree or higher	0.02^*^	0.041	0.008	0.05^***^	<0.01	0.026
Information missing	0.02	0.546	0.381	0.06	0.158	0.300
Equivalised gross household income quintiles						
Lowest quintile (reference category)	−0.02	0.154		0.01	0.563	
2nd quintile	<0.01	0.946	0.428	0.07^*^	0.016	0.222
3rd quintile	−0.03	0.062	0.838	−0.04	0.052	0.096
4th quintile	0.03^*^	0.03	0.002	0.04	0.07	0.413
Highest quintile	0.02	0.322	0.175	0.04^***^	0.002	0.181
Ethnic and immigration background						
Born in EU15 countries (reference category)	<0.01	0.638		0.01	0.213	
Born in A2A8	0.01	0.621	0.890	−0.01	0.847	0.395
Non-white ethnic minorities, born in UK	<0.01	0.891	0.619	0.02	0.446	0.515
Non-white ethnic minorities, born outside UK	−0.01	0.693	0.552	0.04^*^	0.016	0.728
Other	0.01	0.599	0.976	0.03	0.054	0.928
Number of observations	10,517			10,517		
Deprivation level in the neighborhood or LSOA						
Lowest quartile (reference category)	0.01	0.543		0.05^***^	0.004	
2nd quartile	−0.01	0.209	0.229	0.01	0.515	0.081
3rd quartile	−0.01	0.627	0.488	0.03	0.173	0.137
Highest quartile	0.01	0.544	0.848	0.02	0.29	0.041
Proportion of co-ethnic residents in the neighborhood or LSOA						
Lowest quartile (reference category)	−0.02	0.211		<0.01	0.872	
2nd quartile	0.02	0.415	0.237	0.06^***^	0.005	0.037
3rd quartile	0.01	0.643	0.341	0.06^*^	0.011	0.049
Highest quartile	<0.01	0.787	0.683	−0.01	0.629	0.638
Proportion of voters in the parliamentary constituency who voted for UKIP or BNP in the 2015 General Elections						
Lowest quartile (reference category)	<0.01	0.942		0.07^***^	<0.001	
2nd quartile	<0.01	0.969	0.971	0.01	0.570	0.014
3rd quartile	0.03	0.051	0.233	<0.01	0.900	0.004
Highest quartile	−0.04^*^	0.029	0.365	0.01	0.422	0.023
**Number of observations**	10,517			10,517		

a *Physically or verbally attacked in public places in the past 12 months due to one's ethnicity, religion, nationality, language, accent, dress or appearance;*

b *Felt unsafe in public places the past 12 months due to one's ethnicity, religion, nationality, language, accent, dress or appearance*.

*Models estimated using data from Understanding Society Waves 7 & 9 (2015–2018) using logit with longitudinal weights and standard errors estimated after accounting for complex survey design; controls include are general health, FT student and partnership status; + p < 0.10*,

* *p < 0.05*,

** *p < 0.01*,

*** *p < 0.001*.

Beginning with the first hypothesis, we do not find an overall increase in experiences of ethnic and racial harassment in the period following the Referendum in contrast to our expectation in Hypothesis 1. We do, however, find a statistically significant increase in fear of ethnic and racial harassment: the predicted probability of fearing ethnic and racial harassment was 0.07 prior to the Referendum, and increased three percentage points to 0.10 thereafter. Thus, we do find partial support for Hypothesis 1—the expected increase in fear although not in actual harassment levels.

For Hypothesis 2, although we documented the expected higher experiences of fear of ethnic and racial harassment for women in the period prior to the Referendum, we did not find any stronger change in either experiences or fear of ethnic and racial harassment in response to the Referendum by sex or by age. Hypothesis 3 receives some support, however, in that individuals with a university degree or higher did experience a stronger increase in fear following the Referendum than those with less education.

Turning to Hypothesis 4, we test whether we see evidence of a stronger response to the EU Referendum for EU origin minorities in terms of either experiences or fear of ethnic and racial harassment. As can be seen from the statistical tests for variation, there is no difference in the response across the ethnic groups for either outcome; if anything, the increase in fear appears *weaker* for EU origin minorities.

Finally, we examine variation by the community level characteristics stated in Hypothesis 5. We do not find any difference in the experience of ethnic and racial harassment by type of area that ethnic minorities live in, but we find a stronger increase in fear of ethnic and racial harassment among ethnic minorities living in areas of lower deprivation, higher proportion co-ethnic residents and lower proportion of right wing voters. These results are surprising and in contrast to what we expected based on our pre-Referendum results which showed that these are the “safer areas.” It is possible that the 2016 EU Referendum results came as a shock in the safer areas and resulted in an increase of fear.

As a robustness check for temporal variation in changes in response to the Referendum vote over time, we first tested whether our finding of a post-Referendum increase in fear of ethnic and racial harassment would hold if we restricted our sample only to those respondents who replied at least one year after the Referendum. The results, in Table A1, still hold. Next, we partitioned the post-Referendum period into four 6-monthly periods. The results show that the AME of fear of ethnic and racial harassment for each post-referendum period is similar, around 0.03 with a dip to 0.02 during the July-December 2017 period, but these are not statistically significant at the 5% or even the 10% level of significance (*p*-values range from 0.13 to 0.25), possibly due to the smaller sample sizes of each of these categories (see Table A2). As a sensitivity check we re-estimated all models with the alternative degree variable and the results did not change (tables are available upon request from the authors).

## Discussion

In this paper we set out to estimate whether ethnic minorities in England experienced an increase in exposure to and fear of ethnic and racial harassment following the highly politicized, racially charged, and divisive 2016 EU Referendum. We examined the experiences of both the primary targets of the Leave campaign, namely EU origin immigrants, as well as other ethnic minority groups to assess whether this populist electoral victory would be associated with an increase in discriminatory practices even for groups that were not directly targeted. Our nationally representative data captures ethnic and racial harassment that would qualify as hate crime, as well as milder forms of harassment and feelings of unsafety due to ethnic and racial harassment. Unlike existing research that relies on police reports, this paper therefore provides a more comprehensive assessment of changes in hostility toward ethnic minorities following the EU Referendum. Moreover, the large sample size and extensive range of sociodemographic information included in *Understanding Society* allows us to assess both individual and regional variation in changes of experience and fear of harassment following the Referendum. Finally, even one act of harassment can ripple through the wider community resulting in increased levels of fear which as we know has a detrimental effect on mental health (Nandi et al., 2016). By considering fear of ethnic and racial harassment we are able to capture the wider consequences of the Referendum.

Looking at the period 2015–2018, we do not find a statistically significant increase in reports of ethnic and racial harassment (physical or verbal attacks perceived to be due to their race, ethnicity, nationality, language, accent, dress or appearance) in public places among white and non-white ethnic minorities, but we do document an increase in fear of such experiences. While there may have been an increase in hate crimes following the Referendum (Carr et al., 2020), we could not detect a statistically significant increase in the milder forms of harassment (which unlike police recorded hate crimes need not constitute a criminal offense) that we have considered in our paper. It is possible that our study lacks the precision to detect short-term (week-long or month-long) increases in harassment. However, other research has documented how fear of ethnic and racial harassment tracks anti-immigrant prejudice (Spörlein and Schlueter, 2020) and thus it is plausible that the knowledge of this spike in hate crimes combined with ongoing rhetoric targeting immigrants particularly from EU countries could be the explanation for the lasting increase in fear that we find.

While the pre-Brexit period did see an increase in police reports of hate crimes, there was a steady decline in hate crimes as reported in the Crime Survey of England and Wales (Home Office, 2020, p. 19). This discrepancy is possibly due to improvements in recording of hate crimes by the police. Like CSEW data, Understanding Society data also shows a mild decline in proportion of ethnic minorities reporting ethnic and racial harassment before 2015. It is possible that the EU Referendum results halted this decline and thus comparison of the period before and after shows no statistically significant change. Another possible reason for not finding any increase in ethnic and racial harassment in our data, is that the EU referendum led to an increase in online hate crimes (Hardy and Chakraborti, 2020, p. 4) which is not recorded in Understanding Society. A third reason could be that there was an increase in hate crimes reported in institutions and by design this survey under-represents individuals living in institutions (while respondents who move into institutions like care homes and universities are followed, the initial sample is drawn from households only).

Contrary to our expectations, we do not find significantly higher increases in the experience or fear of ethnic and racial harassment among immigrants from EU countries, even though they were the target of the pro-Brexit campaign. We also do not find a stronger increase in fear among demographic groups already pre-disposed to higher levels of fear of crime, namely women or older individuals. The one source of individual level heterogeneity in the association between the Referendum and fear of ethnic and racial harassment is education level: those with a university degree reported a stronger increase in fear of ethnic and racial harassment post-Referendum than less educated minorities. This finding aligns with existing research on the “integration paradox,” which documents greater perceived discrimination among more educated immigrants and minorities (Verkuyten, 2016; Steinmann, 2019). Better educated minorities may have higher expectations of fair treatment, as well as greater contact with the majority population, resulting in more awareness of the rhetoric surrounding the Referendum and a stronger sense of fear arising from it. Our research also aligns with qualitative research on the impact of the Referendum on experiences of hostility and fear among previously privileged EU immigrants, that generally found a stronger increase in feelings of unwelcome and fear for the future rather than actual direct experiences of ethnic and racial harassment (Luthra, 2021).

This paper also examined a range of correlated, but conceptually distinct community –level characteristics as potential moderators of the association between the Referendum and experiences and fear of ethnic and racial harassment. As has been found in previous research (Dustmann et al., 2011; Nandi et al., 2016), we show ethnic and racial harassment is more prevalent in areas with lower minority concentration and higher levels of BNP or UKIP support prior to the EU Referendum. We also show that fear of ethnic and racial harassment is higher in these areas, as well as in areas that suffer higher socioeconomic deprivation. In contrast to emerging research which uses police reports of hate crime (Carr et al., 2020), we do not, however, find a stronger *increase* in reports of ethnic and racial harassment following the Referendum in areas with more prior right wing party support. To the contrary, *increases* in fear following the Referendum were stronger in areas that were previously “safe”: areas with less deprivation, higher minority concentration, and weaker prior right-wing support. Our finding here is corroborated with other recent studies that argue that Brexit changed the perceptions of what was “normal” among individuals who were previously less exposed to anti-immigrant attitudes. For minorities residing in safer areas, the results of the election more closely approximated an unexpected shock, creating a larger shift in fear than among those residing in more Leave or white majority dominated areas where anti-immigrant sentiment was potentially more normalized (Albornoz et al., 2020).

The study we have implemented here takes advantage of the year-round fieldwork structure and broad ethnic and racial harassment and fear measures in *Understanding Society*. Yet our study is not without limitations. First, although we initially planned to treat the Referendum vote as an “Unexpected Event during Survey Design” and employ more stringent causal inference models to examine its effects, the characteristics of the event itself as well as the limitations of our data prevented this. The nature of electoral events themselves threaten the validity of treating the vote as a random shock in an experimental design (Muñoz et al., 2020, p. 187), as electoral events are well-anticipated. Moreover, our respondents are asked to report on their experiences of ethnic and racial harassment over the previous year. To capture pre- and post-treatment to reflect this design, we therefore were forced to test for an increase in ethnic and racial harassment and fear of ethnic and racial harassment from a point 1 year after the EU referendum (Table A1). While our results were robust to this restriction, this is a time point well beyond the event.

Given that other research generally finds a fairly short-lived effect of random events on hate crime (Carr et al., 2020; Schilter, 2020) and attitudinal change (Cappiali et al., 2018; although see also Nägel and Lutter, 2020 for more long term effects), most causal inference models would have required a large number of observations directly before and after the event. Unfortunately, the interview schedule for our sample in *Understanding Society* is insufficiently clustered around the Referendum date (as discussed in the Data section) to provide the statistical power necessary to identify short-term effects.

A second limitation of this study is that, although it encompasses a wider range of behaviors than police reports of hate crime, the survey does not include measures of online ethnic and racial harassment, micro-aggressions^6^ nor of ethnically motivated crimes against property (for instance, defacing immigrant places of worship, businesses or homes). Recent evidence shows an increase in online hate crimes after the EU Referendum (Hardy and Chakraborti, 2020, 2020, p. 4). Third, given the concentration of ethnic minorities in England, we have restricted our analysis to this country alone, and do not observe changes in fear and experiences of ethnic and racial harassment in the other countries of the United Kingdom that experienced the Referendum vote. Finally, as surveys like CSEW and Understanding Society are household surveys, institutional populations are under-represented in the sample.

Despite these shortcomings, there are many offsetting advantages to using a nationally representative, non-self-selected survey. What we can show with our data is a general increase in fear following the Referendum that has endured well-beyond the initial announcement of the election results, and that it varies across different subpopulations. While the spike in hate crimes following the referendum may not have translated into a spike in milder forms of harassment, this spike along with the negative portrayal of immigrants, particularly from EU, may have been instrumental in increasing fear of ethnic and racial harassment among Britain's ethnic minorities—all ethnic minorities not just those who were the target of the Leave campaign. The campaign to Leave the EU with its rhetoric of demonizing migrants and use of phrases like “swarms of immigrants” may have taken a toll on the well-being of Britain's ethnic minorities. Our finding that the Referendum increased fear of ethnic and racial harassment among those who are more privileged and living in safer areas points toward a widespread and perhaps long-lasting increase in fear.

## Data Availability Statement

Publicly available datasets were analyzed in this study. Information about how to access this data can be found here: https://www.understandingsociety.ac.uk/documentation/access-data.

## Author Contributions

AN and RL contributed to conception, design of the study, and both contributed to the Introduction, Data and Methods, Results, and Discussion. AN organized the database and performed the statistical analysis. RL wrote the Background section. Both authors contributed to manuscript revision, read, and approved the submitted version.

## Conflict of Interest

The authors declare that the research was conducted in the absence of any commercial or financial relationships that could be construed as a potential conflict of interest.

## Footnotes

^1^Ethical approval statements are available here.

^2^Most of the interviews took place between January 2015 and December 2018, but if some respondents could not be contacted during the intended fieldwork period, they were contacted and interviewed in the early part of 2019. Throughout this paper we will report the period of study as 2015–2018 as that is when most of the interviews took place.

^3^See Available online at: https://www.nomisweb.co.uk/census/2011/ for England and Wales, https://www.scotlandscensus.gov.uk/ for Scotland and https://www.nisra.gov.uk/publications/2011-census-key-statistics-northern-ireland for Northern Ireland Census 2011 statistics.

^4^Available online at: https://www.electoralcommission.org.uk/media/3016.

^5^Understanding Society provides a variable in each wave, which equals the modified OECD equivalence scale, computed using information on household size and composition and OECD equivalence scale guidelines: Available online at: http://www.oecd.org/els/soc/OECD-Note-EquivalenceScales.pdf.

^6^An attempt has been made to capture this form of everyday racism by the End Everyday Racism project (Available online at: https://racismatcambridge.org).
