# Supplementary material for: The EU Referendum and Experiences and Fear of Ethnic and Racial Harassment: Variation Across Individuals and Communities in England
Source: Front Sociol. 2021 May 14;6:660286. doi: 10.3389/fsoc.2021.660286 (PMC8160085; doi:10.3389/fsoc.2021.660286)
Supplement: Supplementary file 1 [file Table_1.docx]

| Table A1: Average marginal effect (AME) of the EU Referendum on ethnic and racial harassment and fear of such experiences for the whole sample, and separately by sub-groups, excludes June 2016 – May 2017 | | | | | | |
| --- | --- | --- | --- | --- | --- | --- |
|  | Ethnic and racial harassment^1^ | | | Fear of ethnic and racial harassment^2^ | | |
|  | AME | p-value | p-value of difference with the reference category | AME | p-value | p-value of difference with the reference category |
| All | 0.00 | 0.932 |  | 0.02* | 0.039 |  |
| *For different sub-groups* |  |  |  |  |  |  |
| Gender |  |  |  |  |  |  |
| Men (reference category) | <0.01 | 0.841 |  | 0.02 | 0.098 |  |
| Women | <0.01 | 0.827 | 0.763 | 0.02 | 0.124 | 0.942 |
| Age group |  |  |  |  |  |  |
| 16-19 years (reference category) | 0.02 | 0.288 |  | 0.05 | 0.317 |  |
| 20-29 years | 0.01 | 0.685 | 0.571 | 0.06 | 0.064 | 0.582 |
| 30-39 years | <0.01 | 0.829 | 0.335 | <0.01 | 0.952 | 0.375 |
| 40-49 years | -0.01 | 0.471 | 0.184 | <0.01 | 0.94 | 0.366 |
| 50-59 years | -0.01 | 0.748 | 0.385 | 0.04 | 0.205 | 0.913 |
| 60+ years | 0.01 | 0.479 | 0.972 | 0.02 | 0.087 | 0.638 |
| Highest educational qualifications |  |  |  |  |  |  |
| No college degree (reference category) | -0.01 | 0.381 |  | 0.01 | 0.672 |  |
| Received college degree or higher | 0.02 | 0.174 | 0.103 | 0.05^**^ | 0.001 | 0.03 |
| Information missing | 0.02 | 0.74 | 0.626 | 0.04 | 0.399 | 0.519 |
| Equivalised gross household income quintiles |  |  |  |  |  |  |
| Lowest quintile (reference category) | -0.02 | 0.232 |  | <0.01 | 0.963 |  |
| 2nd quintile | 0.02 | 0.422 | 0.187 | 0.07^*^ | 0.034 | 0.144 |
| 3rd quintile | -0.05^**^ | 0.004 | 0.211 | -0.04 | 0.073 | 0.294 |
| 4th quintile | 0.02 | 0.078 | 0.011 | 0.02 | 0.212 | 0.478 |
| Highest quintile | 0.02 | 0.239 | 0.182 | 0.05^**^ | 0.008 | 0.115 |
| Ethnic and immigration background |  |  |  |  |  |  |
| Born in EU15 countries (reference category) | <0.01 | 0.580 |  | 0.01 | 0.265 |  |
| Born in A2A8 | 0.02 | 0.515 | 0.463 | -0.03 | 0.314 | 0.330 |
| Non-white ethnic minorities, born in UK | <0.01 | 0.923 | 0.660 | 0.02 | 0.583 | 0.546 |
| Non-white ethnic minorities, born outside UK | <0.01 | 0.995 | 0.626 | 0.04^*^ | 0.023 | 0.716 |
| Other | <0.01 | 0.865 | 0.674 | 0.02 | 0.141 | 0.823 |
| Number of observations | 7,922 |  |  | 7,922 |  |  |
| ^1^Physically or verbally attacked in public places in the past 12 months due to one's ethnicity, religion, nationality, language, accent, dress or appearance; ^2^Felt unsafe in public places the past 12 months due to one's ethnicity, religion, nationality, language, accent, dress or appearance | | | | | | |
| Models estimated using data from Understanding Society Waves 7 & 9 (2015-18) using logit with longitudinal (self-completion) weights and standard errors estimated after accounting for complex survey design; controls include are general health, FT student and partnership status; + p<0.10 * p<0.05 ** p<.01 | | | | | | |

| Table A1: Average marginal effect (AME) of the EU Referendum on ethnic and racial harassment and fear of such experiences for the whole sample, and separately by sub-groups, excludes June 2016 – May 2017 (continued) | | | | | | |
| --- | --- | --- | --- | --- | --- | --- |
|  | Ethnic and racial harassment^1^ | | | Fear of ethnic and racial harassment^2^ | | |
|  | AME | p-value | p-value of difference with the reference category | AME | p-value | p-value of difference with the reference category |
| Deprivation level in the neighbourhood or LSOA |  |  |  |  |  |  |
| Lowest quartile (reference category) | 0.01 | 0.707 |  | 0.03 | 0.062 |  |
| 2nd quartile | -0.01 | 0.080 | 0.180 | <0.01 | 0.845 | 0.123 |
| 3rd quartile | -0.01 | 0.627 | 0.590 | 0.04 | 0.117 | 0.487 |
| Highest quartile | 0.02 | 0.404 | 0.908 | 0.02 | 0.428 | 0.197 |
| Proportion of co-ethnic residents in the neighbourhood or LSOA |  |  |  |  |  |  |
| Lowest quartile (reference category) | -0.02 | 0.100 |  | -0.01 | 0.729 |  |
| 2nd quartile | 0.02 | 0.358 | 0.153 | 0.07^**^ | 0.007 | 0.010 |
| 3rd quartile | 0.01 | 0.589 | 0.226 | 0.05 | 0.066 | 0.084 |
| Highest quartile | <0.01 | 0.923 | 0.428 | -0.01 | 0.499 | 0.707 |
| Proportion of voters in the parliamentary constituency who voted for UKIP or BNP in the 2015 General Elections |  |  |  |  |  |  |
| Lowest quartile (reference category) | <0.01 | 0.984 |  | 0.08^**^ | 0.001 |  |
| 2nd quartile | 0.01 | 0.61 | 0.801 | 0.02 | 0.362 | 0.014 |
| 3rd quartile | 0.03^*^ | 0.03 | 0.23 | -0.02 | 0.22 | <0.001 |
| Highest quartile | -0.05^**^ | 0.004 | 0.206 | 0.01 | 0.693 | 0.008 |
| Number of observations | 7,922 |  |  | 7,922 |  |  |
| ^1^Physically or verbally attacked in public places in the past 12 months due to one's ethnicity, religion, nationality, language, accent, dress or appearance; ^2^Felt unsafe in public places the past 12 months due to one's ethnicity, religion, nationality, language, accent, dress or appearance | | | | | | |
| Models estimated using data from Understanding Society Waves 7 & 9 (2015-18) using logit with longitudinal weights and standard errors estimated after accounting for complex survey design; controls include are general health, FT student and partnership status; + p<0.10 * p<0.05 ** p<.01 | | | | | | |
