# Supplementary material for: The EU Referendum and Experiences and Fear of Ethnic and Racial Harassment: Variation Across Individuals and Communities in England
Source: Front Sociol. 2021 May 14;6:660286. doi: 10.3389/fsoc.2021.660286 (PMC8160085; doi:10.3389/fsoc.2021.660286)
Supplement: Supplementary file 2 [file Table_2.docx]

| Table A2: Average marginal effect (AME) of the EU Referendum over different periods on ethnic and racial harassment and fear of such experiences for the whole sample | | | | |
| --- | --- | --- | --- | --- |
|  | Ethnic and racial harassment^1^ | | Fear of ethnic and racial harassment^2^ | |
|  | AME | P-values | AME | P-values |
| January 2015 – June 2016 (Reference) |  |  |  |  |
| July - December 2016 | <0.01 | 0.892 | 0.03 | 0.093 |
| January - June 2017 | <-0.01 | 0.986 | 0.03 | 0.126 |
| July - December 2017 | <-0.01 | 0.573 | 0.02 | 0.202 |
| January - June 2018 | 0.01 | 0.470 | 0.03 | 0.128 |
| July - December 2018 | <-0.01 | 0.678 | 0.02 | 0.124 |
|  | 10,517 | | 10,517 | |
| ^1^Physically or verbally attacked in public places in the past 12 months due to one's ethnicity, religion, nationality, language, accent, dress or appearance; ^2^Felt unsafe in public places the past 12 months due to one's ethnicity, religion, nationality, language, accent, dress or appearance | | | | |
| Models estimated using data from Understanding Society Waves 7 & 9 (2015-18) using logit with longitudinal weights and standard errors estimated after accounting for complex survey design; controls include are general health, FT student and partnership status; + p<0.10 * p<0.05 ** p<.01 | | | | |
